# Investigating the determinants of under-five child mortality in Türkiye: the role of inequality in access to healthcare

**DOI:** 10.1186/s12992-025-01182-9

**Published:** 2025-12-24

**Authors:** Selman Kizilkaya, Burhan Durgun, Funda Durgun

**Affiliations:** 1 Faculty of Economics and Administrative Sciences, Department of Health Management, Diyarbakır, Türkiye; 2 Faculty of Economics and Administrative Sciences, Department of Economics, Diyarbakır, Türkiye

**Keywords:** Under-Five mortality, Health expenditures, Health equality, Human capital

## Abstract

**Background:**

Under-five mortality remains a key indicator of healthcare effectiveness, yet disparities persist due to inequitable health investments, and human capital development.

**Aim:**

This study investigates the long-run determinants of under-five mortality in Türkiye by incorporating health expenditures, human capital, and health equality into a robust econometric framework. A key focus is to examine the extent to which health equality modulates the impact of health expenditures on child mortality and whether an inequitable distribution of healthcare services undermines the mortality-reducing effects of financial investments.

**Method:**

This study employs time-series econometric techniques to analyze secondary data from 1975 to 2022. Stationarity is tested using the ADF and KPSS tests, while the Bayer-Hanck cointegration test assesses long-run equilibrium. The FMOLS method estimates the long-term impact of key variables on under-five mortality, addressing autocorrelation and heteroscedasticity concerns.

**Results:**

The results indicate that increased health expenditures and greater human capital reduce under-five mortality, and this pattern remains consistent across both model specifications. When health equality is included in the analysis, its influence emerges as the strongest factor in lowering mortality. However, the interaction between health expenditures and health equality shows that the mortality-reducing effect of health expenditures weakens when resources are distributed unfairly. These findings highlight that the effectiveness of financial investments depends heavily on the degree of equity within the health system.

**Conclusion:**

This study confirms that health expenditure, human capital development, and equitable resource distribution are key to reducing under-five mortality. However, the results also show that when health expenditures are allocated inequitably, their impact becomes weaker, underscoring the critical role of equality. Policies must therefore prioritize fairness in the distribution of health resources, while future research should continue to examine how health expenditures and human capital can be optimized to improve accessibility and efficiency.

## Introduction

Health is a central component of human capital and an essential determinant of productivity, welfare, and social development. The theoretical framework explaining the determinants of health outcomes was first proposed by Grossman, who conceptualized health as both a consumption and an investment good within the human capital model [1]. According to this theory, individuals derive direct utility from being healthy and invest in their health to increase their productive time and lifetime earnings. Health is therefore treated as a durable capital stock that depreciates over time but can be replenished through medical care, education, and behavioral choices. Building on this foundation, Rosenzweig and Schultz extended the analysis to child health, proposing that maternal education, fertility choices, and access to healthcare collectively determine child survival [2]. These models establish a direct theoretical link between socioeconomic factors and child mortality, emphasizing how individual decisions interact with systemic inequalities.

Education plays a particularly profound role in this context. As noted by Becker and by Grossman and Kaestner, education enhances health-related knowledge, resource management, and access to preventive care [3, 4]. Educated mothers, for instance, are more likely to seek prenatal care, practice better hygiene, and ensure timely immunization for their children, resulting in lower under-five mortality rates. This aligns with empirical evidence showing that maternal education is among the most powerful predictors of child survival, particularly in developing countries [5]. However, the relationship between education, income, and health may also be bidirectional—poor health can constrain educational attainment and income potential, perpetuating intergenerational inequalities [6]. Therefore, understanding child mortality requires not only assessing economic inputs but also considering the distributional and institutional dimensions of health systems.

In developing and middle-income countries, including Türkiye, the effectiveness of health investments is closely tied to equality in access and efficiency of resource allocation. Despite overall progress in health indicators, disparities persist between regions and income groups. Studies show that unequal access to healthcare, education, and social security remains a major determinant of child mortality [7, 8]. For example, in Türkiye, eastern provinces exhibit higher under-five mortality rates compared to the western regions, largely due to differences in income, education, and health workforce density [9]. These inequalities reflect the broader structural constraints captured in the Andersen–Newman model, which identifies socioeconomic and institutional barriers as critical determinants of healthcare utilization [10]. Integrating these theoretical and empirical perspectives provides a foundation for analyzing how human capital, health financing, and health equality jointly shape under-five mortality outcomes.

Under-five mortality remains one of the most critical indicators of child health and overall development in a country, yet significant disparities persist across regions and socioeconomic groups. Despite global efforts to reduce under-five mortality, particularly through increasing public health expenditures, the effectiveness of such investments varies widely depending on structural, institutional, and equality-related factors [11]. Numerous studies have explored the role of health expenditures and macroeconomic determinants in reducing child mortality [12], yet empirical findings remain inconclusive. While some research suggests that higher government health expenditures lead to lower mortality rates [13], others indicate that the impact of expenditures is contingent upon governance, efficiency, and equality in distribution [14]. Moreover, recent evidence highlights a strong inverse relationship between current health expenditure and child mortality rates. For instance, findings suggest that every additional US$1 increase in per capita healthcare expenditure corresponds to a reduction of approximately 29,000 under-five child deaths and 19,000 infant deaths. Additionally, neonatal mortality declines by 1.74%, infant mortality by 2.8%, and under-five mortality by 4.6% annually, reinforcing the critical role of sustained health investments in improving child survival rates. However, disparities in health financing remain stark, as seen in India, where per capita healthcare expenditure is the lowest among BRICS nations at approximately US$63, while out-of-pocket expenditure (OOPE) remains disproportionately high at 69.3% [15].

Another key factor influencing under-five mortality is human capital, as investments in education and skill development enhance healthcare awareness, maternal literacy, and overall child well-being. Human capital has been conceptualized as an investment in education and training, acquired by individuals or institutions to enhance productivity and overall well-being [16]. It further consists of knowledge and skills that individuals develop, maintain, and apply to improve economic and social progress [17]. Similarly, human capital is defined as a multidimensional construct, encompassing individual knowledge, skills, competencies, and other capacities that promote personal, societal, and economic development [18]. The role of human capital in reducing under-five mortality rates has been widely documented, particularly in developing regions where investments in education and healthcare infrastructure have led to significant improvements in child health indicators [19]. Empirical evidence from Sub-Saharan Africa highlights the profound impact of human capital accumulation on child survival outcomes. Since 1990, the region has experienced a substantial decline in under-five mortality, largely attributed to improvements in educational attainment, healthcare access, and maternal health literacy. Investments in human capital have also led to an increase in life expectancy at birth, rising from 40 years in 1960 to 62 years in 2019, demonstrating the long-term benefits of sustained educational and health investments [19]. Additionally, primary and secondary school enrollment rates in the region have doubled since 1970, fostering greater health awareness and enabling communities to adopt preventive healthcare practices, which indirectly but significantly contribute to child survival and well-being. Given these empirical trends, continued investment in human capital—particularly through education and healthcare—remains a cornerstone of sustainable child mortality reduction efforts.

From a methodological standpoint, prior studies have primarily relied on cross-sectional or panel data approaches, which may fail to capture the dynamic, long-run relationships between health determinants and child mortality. For example, a study conducted in 2023 aimed to examine the relationship between public health expenditures and under-five mortality rates in low-income countries of Sub-Saharan Africa, providing insights into the effectiveness of healthcare investments in resource-constrained settings [20]. In line with this focus, previous research has examined the role of government health expenditures in reducing under-five mortality in specific national contexts, such as Nigeria, where a significant association between public healthcare spending and child survival rates was found [14]. Additionally, this research seeks to explore the impact of selected national health account indicators on neonatal and under-five mortality across 188 countries from 2000 to 2019, providing a broader perspective on the global dynamics of healthcare financing and child health.

This study contributes to the literature by employing advanced time-series econometric techniques, including stationarity testing, Bayer–Hanck cointegration analysis, and Fully Modified Ordinary Least Squares (FMOLS) estimation, to assess the effects of health expenditures, health equality, and human capital on under-five mortality in Türkiye. By analyzing nearly five decades of data (1975–2022), the research provides a comprehensive understanding of the long-term dynamics shaping child mortality trends. Unlike previous studies that have primarily examined macroeconomic determinants such as GDP per capita or agricultural productivity [21], this study uniquely integrates health equality into the analytical framework to explore its moderating role in the relationship between financial investments and child survival outcomes. The contribution of this study extends beyond the use of econometric tools themselves; it lies in how these models are applied to reveal the structural mechanisms through which inequitable access to healthcare diminishes the effectiveness of health expenditures. By constructing and comparing two complementary models—one examining the direct effects of health expenditures, human capital, and healthcare workforce, and another incorporating the interaction between health expenditures and health equality—the study empirically quantifies the “equality loss” that occurs when resources are unevenly distributed. This approach allows for a nuanced evaluation of how equality conditions influence the efficiency of health investments, moving beyond conventional modeling to provide a theoretically grounded and policy-relevant contribution. In doing so, the paper bridges economic theory and public health policy, offering an innovative framework for understanding how systemic inequalities shape health outcomes and the effectiveness of national health spending.

Given the ongoing global push to reduce child mortality in alignment with the Sustainable Development Goals (SDG 3.2), this study holds substantial policy relevance. Policymakers in emerging economies must not only increase health investments but also ensure that such expenditures are allocated equitably to maximize their impact on child survival [22]. By examining the long-term effectiveness of health policies in Türkiye, this study provides valuable lessons for other developing and middle-income countries seeking to improve child health outcomes through strategic health planning. Ultimately, this research seeks to advance the discourse on sustainable healthcare financing, workforce development, and equality-driven health policies, offering actionable insights for improving child mortality rates globally.

## Materials and methods

This study is theoretically grounded in the Health Capital Model developed by Grossman, which conceptualizes health as both a consumption and an investment good within the framework of human capital theory [1]. According to Grossman’s model, individuals derive direct utility from being healthy and invest in their health to increase productivity and lifetime earnings. Health is treated as a durable capital stock that depreciates over time but can be replenished through medical care, education, and other health-promoting behaviors. In this framework, the health status of a population—measured in this study by the under-five mortality rate (U5MR)—is the outcome of a health production function, where various socioeconomic and institutional factors act as inputs. These include health expenditures, human capital, and equality in access to healthcare. The model assumes that greater investment in these inputs enhances the “production” of health, thereby reducing child mortality.

Building on Grossman’s theoretical foundation [1], subsequent extensions by Rosenzweig and Schultz, Becker, and Wagstaff emphasize that improvements in education, income, and equitable access to healthcare enhance the efficiency of health investments [2, 3, 23]. In the context of Türkiye, this study adopts the health production function framework to examine how inequitable distribution of health expenditures may diminish the mortality-reducing effects of financial investments. By incorporating an interaction term between health expenditures and health equality, the model quantifies the extent to which inequitable access to healthcare undermines the efficiency of health spending, providing a theoretically consistent basis for the empirical analysis presented in the following section. Thus, the theoretical framework integrates Grossman’s health capital model with the concept of equality-adjusted health production, ensuring that the econometric analysis aligns with both human capital theory and contemporary health economics perspectives.

### Data and model

The present study investigates the determinants of under-5 child mortality rates in Türkiye. In accordance with the theoretical framework developed by Grossman, which conceptualizes health as both a consumption and an investment good within human capital theory [1], the variables used in this study were selected to represent the principal components of the health production process. Following Grossman [1], health is conceptualized as both a consumption and an investment good. Individuals derive utility directly from being healthy and invest in health to increase productivity and lifetime earnings. The model can be formally expressed as:

$$\:U=U({H}_{t},{Z}_{t})$$

subject to

$$\:{H}_{t+1}={H}_{t}(1-\delta\:)+{I}_{t}$$.

where $\:{H}_{t}$denotes the stock of health, $\:\delta\:$represents the rate of health depreciation, and $\:{I}_{t}$indicates investment in health through education, healthcare expenditure, and nutrition. At the macroeconomic level, following Boundioa [24], this framework can be expressed as a health production function:

$$\:H=f(E,Y,HE,Env)$$

where $\:H$represents aggregate health status, $\:E$education, $\:Y$income, $\:HE$health expenditure, and $\:Env$environmental factors. This model forms the theoretical foundation of the present study.

The under-five mortality rate (U5MR) serves as the dependent variable because it captures the cumulative effect of economic, social, and institutional factors influencing population health, and is widely recognized as a fundamental indicator of healthcare effectiveness and social development [11, 13, 14]. Health expenditure is included as a key economic input, reflecting the extent of financial investment in the healthcare system and its capacity to enhance child survival outcomes, as demonstrated in both cross-country and country-specific studies [12, 14, 20]. Human capital—measured through education and skills development—captures the long-term effects of literacy, knowledge, and productivity on child well-being, consistent with the frameworks of Becker, Rosenzweig and Schultz, and Grossman and Kaestner [2–4, 16–19]. Finally, health equality is incorporated as a structural and institutional determinant of healthcare accessibility, reflecting evidence that unequal resource distribution and disparities in service provision undermine the mortality-reducing effects of health expenditure [22].

The investigation is based on secondary data sources, and the study period is from 1975 to 2022. In the analyses, human capital, health expenditures, and health equality were employed as explanatory variables. The human capital data, which represent the under-five mortality rate per thousand births and the gross tertiary enrollment ratio, were obtained from the World Bank’s World Development Indicators. Meanwhile, per capita health expenditures were compiled from the OECD database in US dollars (constant 2015). The health equality index, which demonstrates the extent to which healthcare services are provided equally to the society, was obtained from the V-Dem Project database. The study captures healthcare equality through the Health Equality Index provided by the Varieties of Democracy (V-Dem) Project. This index ranges from 0 to 1, where higher values indicate greater fairness in the distribution of healthcare services across the population. The measure reflects the degree to which individuals from different socioeconomic, regional, and demographic groups can access healthcare with similar ease and quality. By incorporating this index into the econometric framework, the analysis captures the structural dimension of healthcare equality and quantifies how disparities in access may influence the efficiency of financial and human resource investments in reducing under-five mortality. In order to minimize the effect of outliers in the data set, the model includes variables other than the health equality index in logarithmic form. First, the effects of health workers, human capital, and health expenditures on under-five mortality rate are analyzed. Empirical model given as follows:

1 $$\:{lu5}_{t}\hspace{0.17em}=\hspace{0.17em}{\beta}_{0}\hspace{0.17em}+\hspace{0.17em}{\beta}_{1}\:{lhc}_{t}\hspace{0.17em}+\hspace{0.17em}{\beta}_{2}\:{lhexp}_{t}\hspace{0.17em}+\hspace{0.17em}{\varepsilon}_{t}\:$$

The model incorporates a series of variables to account for the complex interplay between health equality and health outcomes. Specifically, it includes the under-five mortality rate (*lu5*), and human capital (*lhc*). An interaction variable, derived by multiplying health expenditures by health equality, has been integrated into the model. This interaction variable serves to not only measure the impact of health equality but also to quantify the loss in health expenditures resulting from an inequitable distribution of health services. The interaction term between health expenditures and equality (lhexp×heq) was included to capture the moderating effect of health equality on the efficiency of financial investments, following the conceptual framework proposed by Wagstaff [23] and O’Donnell et al. [25], who emphasized that unequal allocation of health resources may offset the benefits of increased spending. The econometric representation of Model 2, which is formed by adding health equality (*heq*) and the interaction variable (lhexp*heq) to Model 1, is as follows:

2 $$\begin{aligned}\:{lu5}_{t}\hspace{0.17em}&=\hspace{0.17em}{\beta}_{0}\hspace{0.17em}+\hspace{0.17em}{\beta}_{1}\:{lhc}_{t}\hspace{0.17em}+\hspace{0.17em}{\beta}_{2}\:{lhexp}_{t}\hspace{0.17em}\cr&\quad+\hspace{0.17em}{\beta}_{3}\:{heq}_{t}\hspace{0.17em}+\hspace{0.17em}{\beta}_{4}\:{lhexp}_{t}*{heq}_{t}\hspace{0.17em}+\hspace{0.17em}{\varepsilon}_{t}\:\end{aligned}$$

In the models under consideration, *β*_0_ is designated as the autonomous coefficient, while *β*_1_, *β*_2_, *β*_3_, *and β*_4_ represent the elasticities of the independent variables. The error term, designated as εt, is then introduced into the model. Assumptions about elasticities:

- If β_1_, β_2_, β_3_ and β_4_ are equal to 0, a decrease (or increase) in inputs (explanatory variables) has no effect on mortality;
- If β_1_, β_2_, β_3_ and β_4_ are less than 0, an increase (or decrease) in inputs (explanatory variables) leads to a decrease (or increase) in mortality;
- If β_1_, β_2_, β_3_ and β_4_ are greater than 0, an increase (or decrease) in inputs (explanatory variables) leads to an increase (or decrease) in mortality.

The models are subjected to rigorous testing through time series methods. Initially, the degree of integration of the variables is determined by conducting a stationarity test or unit root test. Subsequently, the existence of a long-run relationship is then called into question by conducting a cointegration test. Finally, the long-run parameter estimation of the model is performed. Model 1 provides the baseline specification focusing on the direct effects of health expenditures, and human capital, while Model 2 extends this framework by incorporating health equality and its interaction with expenditures to capture the moderating role of equality. Hence, the two models operate at different analytical levels rather than sequential steps.

### ADF unit root test

Dickey and Fuller, in their study examining the stationarity of time series, assumed that the series follow a first-order autoregressive process [AR(1)] and that the error terms are not autocorrelated [26]. However, this assumption may not always hold. To address cases where the error terms follow autoregressive processes of higher orders, Dickey and Fuller developed the Augmented Dickey–Fuller (ADF) test. The ADF test incorporates lagged values of the dependent variable.

### KPSS stationarity test

The KPSS method, as developed by Kwiatkowski et al., is a statistical test used to evaluate the null hypothesis that an observable series does not contain a unit root but is stationary around a deterministic trend [27]. In contradistinction to the ADF test, the KPSS test adopts the reverse hypothesis and employs the LM (Lagrange Multiplier) test statistic to evaluate stationarity. The model for the test is expressed in Eq. (3).

3 $$\:{y}_{t}=\xi\:t+{k}_{t}+{u}_{t}$$

where $\:{k}_{t}={k}_{t-1}+{\epsilon\:}_{t}$ and$\:{\epsilon\:}_{t}\sim\:WN\left(0,{\sigma\:}_{\epsilon\:}^{2}\right)$. The LM test statistic, denoted in Eq. (4), is computed under the condition $\:{S}_{t}={\sum\:}_{i=1}^{t}{e}_{i}$. The critical values for this test are provided in the study by Kwiatkowski et al. [27].

4 $$\:LM={\sum\:}_{t=1}^{T}{S}_{t}^{2}/{{\widehat{\sigma}}}_{u}^{2}$$

### Bayer-Hanck cointegration test

The test developed by Bayer and Hanck employs meta-analysis to detect the presence of cointegration, a process designed to aggregate quantitative results from independent studies [28, 29]. This approach is comprised of two versions. The first version incorporates the residual-based Engle–Granger test and the system-based Johansen test, while the second version additionally includes the error-correction-based tests of Boswijk and Banerjee et al. [30–33]. The Bayer-Hanck approach is advantageous over other tests in resolving conflicting outcomes among individual test statistics by determining which should be preferred. The test statistic, aggregated using Fisher’s *χ²* test, is calculated in Eq. (5).

5 $$\begin{aligned}&\:EG-JOH-BO-BDM=-2[ln\left({p}_{EG}\right)\cr&\quad+ln\left({p}_{JOH}\right)+ln\left({p}_{BO}\right)+ln\left({p}_{BDM}\right)]\end{aligned}$$

The presence of cointegration necessitates the estimation of long-run coefficients, for which the FMOLS method has been the preferred approach [34]. FMOLS estimator, developed by Phillips and Hansen [34], was employed in this study due to its robustness and efficiency in estimating long-run relationships among cointegrated variables [34]. Unlike conventional Ordinary Least Squares (OLS) estimators, which can produce biased and inconsistent results in the presence of serial correlation and endogeneity, FMOLS applies semi-parametric corrections to both the dependent variable and regressors, thereby ensuring unbiased and consistent long-run parameter estimates [34, 35]. Alternative estimation approaches were also considered. The Dynamic Ordinary Least Squares (DOLS) estimator, proposed by Stock and Watson, corrects endogeneity by including leads and lags of the differenced regressors; however, it often reduces degrees of freedom and may introduce multicollinearity, especially in small-sample studies [36]. Similarly, the Vector Error Correction Model (VECM), introduced by Engle and Granger and further developed by Johansen, primarily captures short-run adjustments and causal dynamics rather than direct long-run elasticities, making it less suitable for the primary objective of this study [30, 31]. In sum, FMOLS was chosen because it produces efficient and consistent long-run elasticity estimates, even with small samples, by addressing endogeneity and serial correlation problems [34, 35]. Since the Bayer–Hanck cointegration test confirmed the presence of a stable long-run relationship among the variables, the FMOLS estimator provides an econometrically sound and theoretically consistent method for analysing the determinants of under-five mortality in Türkiye. FMOLS estimations are obtained from Eq. (6).

6 $$\:{\widehat{\theta\:}}_{FMOLS}=\left[\begin{array}{c}\widehat{\varphi\:}\\\:\widehat{\delta\:}\end{array}\right]=\left({\sum\:}_{t=1}^{T}{Y}_{t}{x}_{t}^{+}-T{\widehat{\lambda\:}}_{12}^{+}\right){\left({\sum\:}_{t=1}^{T}{Y}_{t}{Y}_{t}^{{\prime\:}}\right)}^{-1}$$

where $\:{\widehat{\lambda\:}}_{12}^{+}$ represents the correction term for bias [33].

Another method employed to estimate long-term parameters is the canonical cointegrating regression (CCR), a technique developed by Park [37]. In a manner analogous to the Fully Modified Ordinary Least Squares (FMOLS) method, it corrects endogeneity and deviations by applying stationary transformations to the data [37]. The estimators are calculated using the formula in Eq. (7).

7 $$\:{\widehat{\theta\:}}_{ccr}=\left[\begin{array}{c}\widehat{\beta\:}\\\:\widehat{\gamma\:}\end{array}\right]=\left({\sum\:}_{t=1}^{T}{X}_{t}^{*}{y}_{t}^{*}\right){\left({\sum\:}_{t=1}^{T}{X}_{t}^{*}{{X}_{t}^{*}}^{{\prime\:}}\right)}^{-1}$$

## Results

The descriptive statistics of the variables are presented in Table 1. The distributions of all variables are found to be symmetrical, exhibiting no substantial skewness. The kurtosis values indicate that the variables are flatter (platykurtic) and have thin tails compared to a normal distribution. This finding suggests that there is an absence of extreme outliers. The lowest observed volatility was in health equality, while the highest was in human capital.

**Table 1:** Descriptive statistics

	Mean	Median	Maximum	Minimum	Std. Dev.	Skewness	Kurtosis
*lu5*	3.6919	3.7370	5.0632	2.2618	0.8979	-0.0633	1.6418
*lhc*	3.2036	3.1501	4.8487	1.7144	1.0061	0.2099	1.7813
*lhexp*	6.2374	6.3539	7.3472	5.1020	0.7073	-0.0861	1.4645
*heq*	0.6964	0.6965	0.7938	0.6168	0.0517	0.1644	2.3559
*lhexp×heq*	4.3638	4.4713	5.4727	3.1467	0.7135	-0.2026	1.6745

The stationarity properties of the variables, as presented in Table 2, are analyzed using the Augmented Dickey-Fuller (ADF) unit root test and the Kwiatkowski-Phillips-Schmidt-Shin (KPSS) stationarity test. The ADF test results indicate that all variables are non-stationary at their level form, as their test statistics fail to reject the null hypothesis of a unit root. However, after first differencing, the test statistics become significant at either the 10%, 5%, or 1% levels, confirming that all variables follow an I(1) process. The KPSS test results further support these findings. At level form, all variables, except for the health equality index (heq), are non-stationary at the 1% level, while heq is non-stationary at the 5% level. Following first differencing, the KPSS test statistics fall below the critical values, confirming stationarity. The convergence of results from these two tests validates that all variables are integrated of order one (I(1)), ensuring the appropriateness of further econometric analyses (Table 2).

**Table 2:** ADF and KPSS tests

Variable	ADF I(0)	ADF I(1)	KPSS I(0)	KPSS I(1)
*lu5*	0.8784	-2.7528*	0.9046***	0.2517
*lhexp*	-0.4827	-8.5082***	0.8867***	0.0841
*lhc*	0.6391	-3.8405***	0.8735***	0.1267
*heq*	-2.2381	-5.2986***	0.5054**	0.0875
*lhexp*×*heq*	-1.3862	-4.9020***	0.8280***	0.0813

Notes: *, **, and *** indicate significance at the 10%, 5%, and 1% levels, respectively

The cointegrated relationship between the series stationary at first difference is investigated by Bayer-Hanck cointegration test. As seen in Table 3, the Engle-Granger and the Johansen tests confirm the cointegrated relationship in the estimation of the first model. The composite tests indicated the existence of a cointegration relationship. The second model tests yielded results that demonstrated the variables to be cointegrated, as evidenced by the Johansen, the Banerjee et al. and the Boswijk tests. When the composite test results are analyzed, it is evident that the tests based on two and four tests demonstrate a significant cointegration relationship (Table 3).

**Table 3:** Bayer–Hanck cointegration test results

Model	Engle–Granger	Johansen	Banerjee et al.	Boswijk
**Model 1**				
Statistics	-4.2583	45.5694	-1.9646	9.0329
P-values	0.0104	0.000	0.5447	0.2616
**Fisher Type Tests**	**Statistic**	**10% c.v.**	**5% c.v.**	**1% c.v.**
EG–J	64.39394	8.479	10.895	16.679
EG–J–Ba–Bo	68.29086	16.444	21.106	32.077
**Model 2**				
Statistics	-3.1502	77.3697	-5.1137	86.2843
P-values	0.4934	0.000	0.0018	0.000
**Fisher Type Tests**	**Statistic**	**10% c.v.**	**5% c.v.**	**1% c.v.**
EG–J	56.67491	8.301	10.576	15.845
EG–J–Ba–Bo	124.5769	15.938	20.143	30.774

Note: c.v. denotes critical value

The long-run parameter estimation results, obtained using the FMOLS method, are presented in Table 4 and provide valuable insights into the impact of key health-related variables on under-five mortality. The results obtained from Model 1 indicate that both human capital and health expenditures have a negative impact on child mortality. Additionally, a 1% increase in health expenditures (lhexp) reduces the under-five mortality rate by 0.29%, highlighting the importance of financial investment in health services. A 1% increase in human capital (lhc) also reduces the under-five mortality rate by 0.47%.

The coefficients estimated in Model 2 remain consistent with Model 1 in terms of direction of effect and significance. While the effect of human capital on reducing mortality rates remains almost the same, the effect of health expenditures (lhexp) intensifies. However, the inclusion of the health equity index (heq) causes a significant change in the results. It was found that a one-unit increase in health equity reduces the under-five mortality rate by 11%, reinforcing the idea that a more equitable distribution of health services plays a fundamental role in reducing child mortality. A particularly striking finding emerges from the interaction term (lhexp*heq), which captures the combined effect of health expenditures and equity. The coefficient of this interaction term is positive and statistically significant (1.7483; *p* < 0.05), indicating that the potential life-saving effect of health expenditures weakens or even reverses when they are distributed unfairly. This implies that while increased health expenditures are generally beneficial, their effectiveness depends on how fairly they are distributed across the population.

The high R² values (0.9840 in Model 1 and 0.9858 in Model 2) indicate that the estimated models exhibit strong explanatory power, capturing nearly all the variations in under-five mortality. The adjusted R² values further confirm the robustness of the model specification. In conclusion, the FMOLS estimates affirm that health expenditures and workforce expansion are essential in reducing child mortality. However, the results also highlight the critical role of health equality in ensuring that financial investments translate into meaningful health outcomes (Table 4).

**Table 4:** Long-run estimates results (FMOLS)

Variable	Coefficient (Model 1)	t-Statistic (Model 1)	Coefficient (Model 2)	t-Statistic (Model 2)
*c*	8.8776**	17.6887	16.6266**	4.8216
*lhc*	-0.4780**	-5.6595	-0.4978**	-6.8874
*lhexp*	-0.5859**	-4.8558	-1.7442**	-3.1674
*heq*	—	—	-11.6218*	-2.2664
*lhexp*×*heq*	—	—	1.7483*	2.1626
*R²*	0.9840		0.9858	
*Adjusted R²*	0.9832		0.9844	

Notes: * and ** indicate significance at the 5%, and 1% levels, respectively

The robustness checking of the long-term coefficients was tested using the CCR method. The findings presented in Table 5 are consistent with those obtained using the FMOLS approach. While the sign of the obtained coefficients is consistent, there are negligible discrepancies in the magnitude of the coefficients (Table 5).

**Table 5:** Robustness check (CCR)

Variable	Coefficient (Model 1)	t-Statistic (Model 1)	Coefficient (Model 2)	t-Statistic (Model 2)
*c*	8.8816**	17.8473	16.4915**	4.9414
*lhc*	-0.4769**	-5.6067	-0.5045**	-6.8582
*lhexp*	-0.5869**	-4.9012	-1.7206**	-3.2253
*heq*	—	—	-11.4715*	-2.3008
lhexp*heq	—	—	1.7260*	2.1934
*R* ^*2*^	0.9839		0.9858	
*Adjusted R* ^*2*^	0.9832		0.9844	

Notes * and ** indicate significance at the 5% and 1% levels, respectively

## Discussion

The findings of this study contribute significantly to the ongoing discourse on the determinants of under-five mortality by integrating health expenditures, human capital, and health equality into a long-run econometric framework. The results confirm that while increased health expenditures reduce child mortality, their effectiveness is largely dependent on equality in resource allocation. The study further highlights the paradox of healthcare spending, wherein financial investments alone are insufficient in achieving meaningful reductions in under-five mortality unless accompanied by structural and institutional improvements in healthcare delivery.

The significant negative relationship between health expenditures and under-five mortality aligns with existing literature suggesting that higher public health investments contribute to better child health outcomes [35, 38]. However, the findings of this study emphasize that the distributional aspect of these expenditures is crucial. The interaction term between health expenditures and health equality, which yields a positive coefficient, suggests that when health expenditures are not equitably allocated, their mortality-reducing effects are weakened or even reversed. This is consistent with recent studies indicating that health financing reforms must incorporate equality-based approaches to be effective [39]. While these findings highlight the importance of equality in healthcare spending, the broader literature presents mixed evidence on the effectiveness of health expenditures in reducing child mortality. For instance, several studies found a significant and positive effect of health expenditure on under-five mortality in BRICS countries, Nigeria, and Malaysia, respectively [14, 40, 41]. These results suggest that higher health expenditures alone do not guarantee better child health outcomes, as factors such as governance, resource misallocation, and inefficiencies in service delivery may offset their intended benefits. Additionally, studies examining the impact of health expenditures on under-five mortality in SSA have primarily focused on public health expenditure as the main indicator of health spending, often overlooking the role of private [42, 43], out-of-pocket [44], and external health expenditures [33]. Some studies assessed the influence of public and private health expenditures on health outcomes in SSA countries using a fixed-effects estimation technique, concluding that while public health expenditure significantly reduces under-five mortality, private health expenditure appears to be insignificant in this context [42, 43]. Similarly, other research found that public and external health expenditures contribute positively to child health outcomes, whereas private health expenditure has a negligible impact [45]. These contrasting findings underscore the complexity of the relationship between health expenditures and under-five mortality, suggesting that financial investments in healthcare must be accompanied by equitable distribution, effective governance, and strategic resource allocation to yield meaningful reductions in child mortality.

Human capital was also found to play a crucial role in shaping child mortality outcomes. This suggests that while higher education levels and health literacy contribute to improved child health, other factors such as economic conditions, healthcare accessibility, and maternal employment may moderate its impact. Prior studies have demonstrated that higher maternal education leads to improved health-seeking behaviors and better nutritional practices, thereby reducing child mortality [46]. Some countries have successfully leveraged educational expansion to improve child health, others continue to experience high child mortality rates despite rising education levels, due to persistent economic and healthcare access barriers [22].

The findings also shed light on the complex relationship between healthcare infrastructure and child mortality reduction efforts. While some studies suggest that proximity to healthcare facilities is a key determinant of improved child health outcomes, this study did not find a statistically significant effect of healthcare infrastructure alone on under-five mortality [47]. This is consistent with research in Malawi, where reducing the distance to health facilities did not necessarily translate into improved child survival, due to persistent barriers related to healthcare quality, transportation access, and economic affordability [48]. These results suggest that simply expanding physical healthcare infrastructure is insufficient, and that service delivery efficiency and affordability must be considered in parallel.

## Conclusion

This study provides a comprehensive econometric analysis of the determinants of under-five mortality in Türkiye, focusing on the long-run effects of health expenditures, human capital, and health equality. By employing time-series econometric techniques, including stationarity tests, cointegration analysis, and FMOLS estimation, the study establishes the presence of a stable long-run equilibrium relationship between these variables. The findings highlight that health expenditures significantly contribute to reducing under-five mortality, confirming the importance of financial investment and human resources in the healthcare sector. However, a key revelation of this study is the crucial role of health equality in shaping child survival outcomes. While increased health expenditures are generally associated with a reduction in under-five mortality, the interaction between health expenditures and equality produces a positive coefficient, suggesting that when healthcare investments are allocated inequitably, their beneficial effects may be diminished or even negated. This finding underscores the paradoxical nature of health spending, emphasizing that increased expenditures alone do not necessarily translate into better health outcomes unless accompanied by mechanisms ensuring equitable distribution.

Furthermore, the insignificance of human capital in both models suggests that improvements in education and knowledge accumulation alone may not be sufficient to drive reductions in child mortality unless they are coupled with structural healthcare improvements. This outcome highlights the need for an integrated policy approach, wherein financial resources, medical workforce capacity, and equitable access to healthcare services are jointly considered in the formulation of effective child mortality reduction strategies.

The study’s findings hold significant implications for health policymakers, as they indicate that merely increasing health expenditures without addressing systemic inequalities in healthcare accessibility may lead to suboptimal outcomes. The results reinforce the imperative of equality-driven healthcare investments to ensure that financial allocations effectively translate into improved health conditions, particularly for vulnerable populations.

### Practical implications

The findings of this study offer profound practical implications for health policymakers, international organizations, and public health administrators striving to reduce under-five mortality through evidence-based interventions. The results confirm that health expenditures contribute to reducing child mortality, but only when equitable healthcare distribution mechanisms are in place. The positive interaction effect between health expenditures and equality suggests that when healthcare investments are unevenly distributed, the effectiveness of financial inputs is compromised. This finding necessitates the reallocation of healthcare resources to ensure that underserved populations receive adequate access to medical services, particularly in rural and economically disadvantaged areas. Moreover, the study highlights the limitations of human capital alone in explaining reductions in under-five mortality. While education and knowledge accumulation remain essential for improving public health awareness, they do not substitute for direct investments in medical infrastructure and healthcare accessibility. Policymakers should adopt multidimensional health strategies that integrate educational programs, medical workforce development, and equitable financial investments to achieve sustainable improvements in child health outcomes. From an international perspective, the findings emphasize the importance of equality-driven health policies in low- and middle-income countries, where health disparities often exacerbate child mortality rates. Global health organizations and policymakers should design targeted intervention programs that incorporate both financial investment and equitable healthcare access, ensuring that vulnerable populations benefit from public health expenditures.

### Limitations

The study acknowledges several substantial limitations that may influence the interpretation of its findings. The analysis is confined to Türkiye, which limits the external validity of the results and their applicability to other countries with different institutional and healthcare structures. Moreover, the use of secondary data over a long historical period may involve measurement inconsistencies and unobserved structural changes. Although these factors could constrain the precision of estimates, the application of robust time-series techniques, including cointegration and FMOLS, mitigates potential biases and supports the reliability of long-run relationships. Institutional variables such as governance quality and policy efficiency were not directly included in the model, which could further refine future analyses. Despite these constraints, the consistency of results across models and the strong statistical fit indicate that the main conclusions remain credible and robust. Future research should nevertheless test these relationships across different contexts and with alternative methodologies to strengthen external validity.

### Future directions for research

Given the profound policy implications derived from this study, several avenues for future research emerge. Future studies should expand the geographical scope of analysis by conducting cross-country comparisons to examine whether the moderating role of health equality in shaping under-five mortality outcomes holds across different healthcare systems. Such comparative analyses could provide global insights into the effectiveness of health investments in diverse economic and institutional contexts. Moreover, further research should explore the short-run dynamics of health expenditures and equality by integrating vector error correction models or time-varying parameter models to capture potential nonlinearities and structural breaks in the relationship between health spending and child mortality. Additionally, future investigations should incorporate micro-level health data to assess how household-level healthcare access, nutrition, and socioeconomic factors interact with macro-level expenditures and workforce density in determining child mortality rates. Lastly, given the growing role of digital health technologies and telemedicine, future research should examine how technological advancements in healthcare delivery impact child mortality rates. Exploring the potential of digital health solutions, mobile health applications, and remote medical services could offer valuable insights into innovative strategies for improving healthcare access and equality in both developed and developing economies.

## Data Availability

The datasets analyzed during the current study are obtained from publicly available and reputable sources, including the World Bank, the World Health Organization (WHO), and the Varieties of Democracy (V-Dem) Project.

## References

[1] Grossman M. On the concept of health capital and the demand for health. J Polit Econ. 1972;80(2):223–55.

[2] Rosenzweig MR, Schultz TP. The behavior of mothers as inputs to child health. In: Fuchs V, editor. Economic aspects of health. Chicago: University of Chicago Press; 1983. pp. 53–92.

[3] Becker GS. Human capital: A theoretical and empirical Analysis, with special reference to education. Chicago: University of Chicago Press; 1964.

[4] Grossman M, Kaestner R. Effects of education on health. In: Behrman J, Stacey N, editors. The social benefits of education. Ann Arbor: University of Michigan Press; 1997. pp. 69–123.

[5] Kiross GT, Chojenta C, Barker D, Loxton D. The effect of maternal education on infant mortality in ethiopia: a systematic review and meta-analysis. PLoS ONE. 2019;14(7):e0219578. 31356599 10.1371/journal.pone.0220076 PMC6663004

[6] Aghion P, Howitt P. Endogenous growth theory. Cambridge (MA): MIT Press; 1998.

[7] Wagstaff A. Socioeconomic inequalities in health: cross-country evidence. Soc Sci Med. 2003;57(9):1529–38. 12948564 10.1016/s0277-9536(02)00555-5

[8] World Health Organization. World report on social determinants of health equality. Geneva: WHO; 2025.

[9] Pala K. Türkiye’de sağlık alanında eşitsizlikler. İstanbul: İstanbul Politik Araştırmalar Enstitüsü; 2021.

[10] Andersen R, Newman JF. Societal and individual determinants of medical care utilization in the united States. Milbank Q. 1973;51(1):95–124. 4198894

[11] Osei B, Kulu E, Appiah-Konadu P. Government health expenditure and child health: empirical evidence from West African countries. Int J Soc Econ. 2024;51(6):786–99. 10.1108/IJSE-03-2022-0212.

[12] Agbatogun KK, Opeloyeru OK. Macroeconomic determinants of under-five mortality rate in Nigeria. Signifikan. 2020;9(2):177–86. 10.15408/sjie.v9i2.13751.

[13] Sibanda K, Qoko A, Gonese D. Health expenditure, institutional quality, and under-five mortality in Sub-Saharan African countries. Int J Environ Res Public Health. 2024;21(3):333. 10.3390/ijerph21030333. 38541331 10.3390/ijerph21030333 PMC10970304

[14] Azuh DE, Osabohien R, Orbih M, Godwin A. Public health expenditure and under-five mortality in nigeria: an overview for policy intervention. Open Access Maced J Med Sci. 2020;8(E):353–62. 10.3889/oamjms.2020.4327.

[15] Sridevi M, Laxmaiah A. Healthcare financing and under-5 child mortality among the BRICS nations. Indian J Hum Dev. 2020;14(3):507–17. 10.1177/0973703020976717.

[16] Alika IJ, Stan A. Human capital: definitions, approaches and management dynamics. J Bus Admin Educ. 2014;5(1):55–78.

[17] Armstrong M. Armstrong’s handbook of human resource management practice. 15th ed. London: Kogan Page; 2019.

[18] Murtin F, Mackenbach J, Jasilionis D, Mira D. Educational inequalities in longevity in 18 OECD countries. J Demogr Econ. 2022;88(1):1–29. 10.1017/dem.2021.22.

[19] UNESCO. Lower secondary completion rate, Sub-saharan Africa. UNESCO Institute for Statistics. Published September; 2021.

[20] Ayipe FI, Tanko M. Public health expenditure and under-five mortality in low-income Sub-Saharan African countries. Soc Sci Humanit Open. 2023;8:1–7. 10.1016/j.ssaho.2023.100570.

[21] Gershon O, Akhigbemidu A, Osabohien RA. Domestic resource mobilization and under-five mortality in Nigeria. Res World Econ. 2020;11(3):320–32. 10.5430/rwe.v11n3p320.

[22] Zewudie AT, Gelagay AA, Enyew EF. Determinants of under-five child mortality in ethiopia: analysis using Ethiopian demographic health survey, 2016. Int J Pediatr. 2020;2020:7471545. 10.1155/2020/7471545. 33029153 10.1155/2020/7471545 PMC7527934

[23] Wagstaff A. Poverty and health sector inequalities. Bull World Health Organ. 2002;80(2):97–105. 11953787 PMC2567730

[24] Boundioa J. Does public indebtedness matter in the effect of public health expenditure on human longevity in Sub-Saharan Africa countries? Evidence from dynamic panel threshold regression. Health Econ Rev. 2025;15:79. 10.1186/s13561-025-00673-0. 41066006 10.1186/s13561-025-00673-0 PMC12512314

[25] O’Donnell O, van Doorslaer E, Wagstaff A, Lindelow M. Analyzing health equality using household survey data: a guide to techniques and their implementation. Washington, DC: The World Bank; 2008.

[26] Dickey DA, Fuller WA. Distribution of the estimators for autoregressive time series with a unit root. J Am Stat Assoc. 1979;74(427):427–31.

[27] Kwiatkowski D, Phillips PC, Schmidt P, Shin Y. Testing the null hypothesis of stationarity against the alternative of a unit root: how sure are we that economic time series have a unit root? J Econometrics. 1992;54(1–3):159–78. 10.1016/0304-4076(92)90104-Y.

[28] Bayer C, Hanck C. Is double trouble? How to combine cointegration tests. Ruhr Econ Pap. 2008. 10.26481/umamet.2008014. (48).

[29] Bayer C, Hanck C. Combining non-cointegration tests. J time Ser Anal. 2012;34(1):83–95. 10.1111/j.1467-9892.2012.00814.x.

[30] Engle RF, Granger CW. Co-integration and error correction: representation, estimation, and testing. Econometrica. 1987;55(2):251–76. 10.2307/1913236.

[31] Johansen S. Statistical analysis of cointegration vectors. J Econ Dyn Control. 1988;12(2–3):231–54. 10.1016/0165-1889(88)90041-3.

[32] Boswijk HP. Testing for an unstable root in conditional and unconditional error correction models. J Econometrics. 1994;63:37–60. 10.1016/0304-4076(93)01560-9.

[33] Banerjee A, Dolado JJ, Mestre R. Error-correction mechanism tests for cointegration in a single-equation framework. J time Ser Anal. 1998;19(3):267–83. 10.1111/1467-9892.00091.

[34] Phillips PC, Hansen BE. Statistical inference in instrumental variables regression with I(1) processes. Rev Econ Stud. 1990;57(1):99–125. 10.2307/2297545.

[35] Şanlısoy S, Kök R. Politik istikrarsızlık- Ekonomik büyüme ilişkisi: Türkiye örneği (1987–2006). Dokuz Eylül Üniversitesi İktisadi ve İdari. Bilimler Fakültesi Dergisi. 2010;25(1):101–25.

[36] Stock JG, Watson MW. A simple estimator of cointegrating vectors in higher order integrated systems. Econometrica. 1993;61(4):783–820.

[37] Park CY. Canonical Cointegrating Regressions Ekonometrica. 1992;60(1):119–43.

[38] Rahman MM, Khanam RM. Health care expenditure and health outcome nexus: new evidence from abstract. Glob Health. 2018;14:1–30. 10.1186/s12992-018-0430-1. 10.1186/s12992-018-0430-1 PMC6249744 30466452

[39] Boachie MK, Põlajeva T, Frimpong AO. Infant mortality in low- and middle-income countries: does government health spending matter? J Dev Policy Pract. 2020;5:54–73. 10.1177/2455133320909916.

[40] Chinnaiyan S, Babu B, Ghimire A. Impact of under-five mortality on economic growth and health-care expenditures in India. MGM J Med Sci. 2021;8(3):227–31. 10.4103/mgmj.mgmj_6_21.

[41] Kulkarni L. Health inputs, health outcomes and public health expenditure: evidence from the BRICS countries. Int J Appl Econ. 2016;23:72–84.

[42] Logarajan RD, Nor NM, Sirag A, Said R, Ibrahim S. The impact of public, private, and out-of-pocket health expenditures on under-five mortality in Malaysia. Healthc (Basel). 2022;10(3):589. 10.3390/healthcare10030589. 10.3390/healthcare10030589 PMC8953126 35327065

[43] Ashiabi N, Nketiah-Amponsah E, Senadza B. The effect of health expenditure on selected maternal and child health outcomes in Sub-Saharan Africa. Int J Soc Econ. 2016;43(12):1386–99. 10.1108/IJSE-08-2015-0199.

[44] Arthur E, Oaikhenan HE. The effects of health expenditure on health outcomes in Sub-Saharan Africa (SSA). Afr Dev Rev. 2017;29(3):524–36. 10.1111/1467-8268.12287.

[45] Ssozi J, Amlani S. The effectiveness of health expenditure on the proximate and ultimate goals of healthcare in Sub-Saharan Africa. World Dev. 2015;76:165–79. 10.1016/j.worlddev.2015.07.010.

[46] Sasmoko S, Handayani W, Nassani AA, Haffar M, Zaman K. Do precarious female employment and political autonomy affect the under-5 mortality rate? Evidence from 166 countries. PLoS ONE. 2022;17(6):e0269575. 10.1371/journal.pone.0269575. 35759457 10.1371/journal.pone.0269575 PMC9236242

[47] Avelino IC, Van-Dúnem J, Varandas L. Under-five mortality and associated risk factors in children hospitalized at David Bernardino pediatric hospital (DBPH), angola: a hierarchical approach. Int J Environ Res Public Health. 2024;21(8):1062. 10.3390/ijerph21081062. 39200671 10.3390/ijerph21081062 PMC11354039

[48] Quattrochi JP, Hill K, Salomon JA, Castro MC. The effects of changes in distance to nearest health facility on under-5 mortality and health care utilization in rural Malawi, 1980–1998. BMC Health Serv Res. 2020;20(1):899. 10.1186/s12913-020-05738-w. 32972395 10.1186/s12913-020-05738-w PMC7517642

